# The SNP rs961253 in 20p12.3 Is Associated with Colorectal Cancer Risk: A Case-Control Study and a Meta-Analysis of the Published Literature

**DOI:** 10.1371/journal.pone.0034625

**Published:** 2012-04-11

**Authors:** Xiawen Zheng, Li Wang, Yaowu Zhu, Qing Guan, Huijun Li, Zhigang Xiong, Lingyan Deng, Jie Lu, Xiaoping Miao, Liming Cheng

**Affiliations:** 1 Department of Laboratory Medicine, Tongji Hospital, Tongji Medical College, Huazhong University of Science and Technology, Wuhan, China; 2 MOE Key Lab of Environment and Health, Department of Epidemiology and Biostatistics, School of Public Health, Tongji Medical College, Huazhong University of Science and Technology, Wuhan, China; 3 Department of Epidemiology and Biostatistics, Institute of Basic Medical Sciences, Chinese Academy of Medical Sciences; School of Basic Medicine, Peking Union Medical College, Beijing, China; National Cancer Center Research Institute, Japan

## Abstract

**Background:**

Colorectal cancer (CRC) is the third common cancer and the fourth leading cause of cancer death worldwide. A single nucleotide polymorphism (SNP), rs961253 located in 20p12, was firstly described to be associated with the increased risk of CRC in a genome-wide association study; however, more recent replication studies yielded controversial results.

**Methodology/Principal Findings:**

A hospital-based case-control study in a Chinese population was firstly performed, and then a meta-analysis combining the current and previously published studies were conducted to explore the real effect of rs961253 in CRC susceptibility. In the Chinese population including 641 cases and 1037 controls, per-A-allele conferred an OR of 1.60 (95% CI = 1.26–2.02) under additive model. In the meta-analysis including 29859 cases and 29696 controls, per-A-allele have an OR of 1.13 (95% CI = 1.09–1.18) under a random-effects model due to heterogeneity (*P* = 0.019). Nevertheless, the heterogeneity can be totally explained by ethnicity, with the *tau^2^*reduced to 0 after including ethnicity in meta-regression model. In stratified analysis by ethnicity, per-A-allele had ORs of 1.34 (95% CI = 1.20–1.50) and 1.11 (95% CI = 1.08–1.14) for Asian and European, respectively, without heterogeneity. Modest influence of each study was observed on overall estimate in sensitive analysis, and evident tendency to significant association was seen in cumulative analysis over time, together indicating the robust stability of the current results.

**Conclusions/Significance:**

The results from our study and the meta-analysis provided firm evidence that rs961253 significantly contributed to CRC risk in both Asian and European population.

## Introduction

Colorectal cancer (CRC) is the third common malignancy and the fourth leading cause of cancer mortality in the world, with more than 1.2 million incidences worldwide each year and approximately 630,000 death from CRC annually [1]. CRC is a complex trait influenced by environmental and genetic factors and their interactions. Analysis of phenotype concordance in twins reflected the strong genetic component to development of CRC [2], which is responsible for ∼35% of all CRC. Nevertheless, high-risk germline mutations in a few genes, such as *APC*, the mismatch repair (*MMR*) genes, *SMAD4*, and*BMPR1A*, explained <5% of total CRC [3]. Growing evidence from epidemiological studies has invoked the common allele-common disease paradigm in CRC. Recent genome-wide association (GWA) studies have validated this hypothesis and implicated multiple common single nucleotide polymorphisms (SNPs) contributing to CRC susceptibility [4]–[12]. Among these SNPs, rs961253 (20p12.3), located in proximity of the gene bone morphogenetic protein 2 (*BMP2*), was firstly identified by Houlston et al. to be significantly associated with CRC risk in the meta-analysis of two GWAS comprising 6780 cases and 6843 controls [5]. BMP2, as a key member of transforming growth factor-beta (TGF-β) super family, has been shown to inhibit colonic epithelial cell growth and promote apoptosis and thus critically involve in development of CRC [13]. However, in the more replication studies results have been inconsistent [14], [15]. In part, the difficulty of replication occurs potentially due to the modest effect of this SNP, with an odds ratio (OR) of 1.12 reported for the minor allele; hence, small genetic association studies have a lack of power and might fail to replicate this association. Additionally, due to the phenomena “winner's curse” that OR of disease variant is usually overestimated in the initial positive study, the necessary sample size of replication study would be underestimated if basis on the initially reported OR, then the replication would be underpowered and possibly fail [16]. Nevertheless, meta-analysis, a statistical tool for combing data across studies, is powerful to clarify inconsistent findings in genetic association studies due to its exponential increase in sample size [17]. Therefore, in this study, we conducted a meta-analysis, combining results from published literature and our case-control study herein performed in a Chinese population, to provide a more precise estimation of the association between rs961253 and CRC risk.

## Materials and Methods

### Study population

A total of 641 incident cases of CRC and 1037 controls were enrolled between 2009 and 2011 from Tongji Hospital of Huazhong University of Science and Technology (HUST), Wuhan, China. All of the subjects were unrelated ethnic Han Chinese in Wuhan region. Cases have been histological confirmed with primary CRC and had not received treatment prior to blood samples collection. Controls were cancer-free individuals randomly selected from a health check-up program at the same Hospital in the same period as the cases were enrolled. Controls were frequency-matched to cases by age (±5 years) and gender. At recruitment, a 5-ml peripheral blood sample was collected from each subject after written informed consent was obtained. This study was approved by the Institutional Review boards of Tongji Hospital of HUST.

### Genotyping

Genomic DNA was extracted from 5-mL of peripheral blood sample using the Relax Gene Blood DNA System DP319-02 (Tiangen, Beijing, China) according to the manufacturer's instructions. The genotypes of rs961253 SNP was determined by the TaqMan SNP Genotyping Assay(Applied Biosystems, Foster city, CA) using the 7900HT Fast Real-Time PCR System (Applied Biosystems, Foster city, CA). For quality control, 5% duplicated samples were randomly selected for to assess the reproducibility, with a concordance rate of 100%.

### Statistical Analysis

The *χ^2^*test, Fisher exact test, and *t* test were applied to estimated differences in the distribution of demographic characteristics and genotypes between cases and controls, where appropriate. Hardy-Weinberg equilibrium (HWE) was assessed by the goodness-of-fit χ^2^test for genotypes in the control group. Under multivariate logistic regression model, the genotypic OR and its 95% CI were calculated after adjusting for age and sex, with the reference of the common homozygote. To avoid the assumptions of genetic models, additive and dominant model for rs961253 in associated with CRC were also analyzed. All above statistical analysis were carried out in the SPSS V12.0.

### Meta-analysis of rs961253 in association with CRC risk

To confirm the involvement of rs961253 in CRC susceptibility, a meta-analysis combining published studies and our case-control study was conducted. We searched the all publications updated to October of 2011 from the PubMed, EMBASE, and ISI Web of Science data bases without language restriction, using the search strategy based on the terms ‘rs961253, BMP2 or 20p12.3’ in combination with ‘Colorectal neoplasmor colorectal cancer’. References listed in retrieved articles were also checked for missing information. The inclusion criteria were: (1) case-control or nested case-control study assessing the association between rs961253and CRC risk; (2) providing data for calculating genotypic odds ratio (ORs) with corresponding 95% confidence interval (95% CI); (3) genotypes in controls being in Hardy-Weinberg equilibrium (*P*>0.01). Animal studies, reviews, simply commentaries and case reports were excluded. Study overlapping with other studies should be eliminated, and the one with larger sample size was selected. If more than one geographic or ethnic population were included in one report, each population was considered separately.

The following data were extracted from each study: first author's name, year of publication, study design, geographic location or ethnicity of study population, control source, sample size, genotyping method, male/female rate, mean age, frequencies of genotypes in cases and controls. Hardy-Weinberg equilibrium in controls was estimated again in the meta-analysis by the goodness-of-fit χ^2^ test (*P*>0.01). Pooled frequency of the A allele in various ethnic populations was estimated using the inverse variance method previously described by Thakkinstian et al. [18].ORs and 95% CIs as the metrics of effect size were re-calculated for the genotypes AA versus CC and CA versus CC. A dominant genetic model was assumed for the rs961253, and an additive “per-allele” model was also considered. The per-allele OR of the A allele was estimated by assigning scores of 0, 1, and 2 to the genotypes CC, CA, and AA, respectively, and calculating ORs per units score by logistic regression model. Between-study heterogeneity across all eligible comparisons was estimated by the Cochran's *Q* statistic and the *I^2^*metric. Heterogeneity was considered significant at *P*<0.10 for the Q statistic [19]. For the *I^2^* metric, the following cut-off points were used: *I^2^* = 0–25%, no heterogeneity; *I^2^* = 25–50%,moderate heterogeneity; *I^2^* = 50–75%, large heterogeneity; *I^2^* = 75–100%, extreme heterogeneity [20]. A fixed-effects model, using Mantel-Haenszel method [21], was applied to pool data from studies when heterogeneity was negligible based on *P* for Q statistic greater than 0.1; otherwise, a random-effects model, using DerSimonian and Laird method [22], was applied. To explore sources of heterogeneity across studies, a meta-regression model was employed [23]. The pre-specified characteristics for assessment of heterogeneity sources were: ethnicity of population (Asian and European), source of control (population and hospital based controls), study type (replication and GWA studies), sample size (≤2000 and >2000 subjects) and genotyping method (high-throughput and low-throughput assays). Stratified analysis was then conducted, according to the potential sources of heterogeneity reported by meta-regression analysis. Sensitivity analysis was conducted to assess influence of each study on overall estimate [24]. Cumulative analysis was performed by assortment of publication times [25]. Publication bias was assessed by funnel plot [26], Egger's test [27], and the trim and fill method [28], which estimates the number and outcomes of potentially missing studies resulting from publication bias. All statistical analyses were carried out in STATA V11.0, and all *P* values are two-tailed with a significant level at 0.05.

## Results

### Case-control study results

#### Population characteristics

A total of 641 incident cases of colorectal cancer and 1037 frequency-matched controls were enrolled in this study. As shown in Table 1, males were 59.9% among cases compared with 59.1% among controls. Mean age was 56.31 years (±12.59) for cases and 57.24 years (±10.86) for controls. There was no significant difference in distribution of sex (*P* = 0.748) and age (*P* = 0.119) between case and control group.

**Table 1:** The characteristics of the study population.

Variables	Cases (N = 641)No. (%)	Controls (N = 1037)No. (%)	*P*
Sex			0.748†
Males	384 (59.9)	613 (59.1)	
Female	257 (40.1)	424 (40.9)	
Age (years)	56.31±12.59	57.24±10.86	0.119‡

† *P* value was calculated by the *χ^2^* test;

‡ *P* value was calculated by the *t* test.

#### Association analysis

Table 2 displays the distribution of rs961253 genotypes in cases and controls. Genotypes in controls were in agreement with Hardy-Weinberg equilibrium (*P* = 0.277). Significant difference was observed in distribution of genotypes between cases and controls (*χ^2^* = 16.33, *P*<0.001). In multivariate regression model, the carriers of the CA genotype showed a significant increased CRC risk as compared with those carrying the CC genotype (OR = 1.56, 95%CI = 1.21–2.01). Due to the low frequency of the AA genotype in this study population, a dominant model was perform, by combining the AA with the CA into an A carrier (AA plus CA) group, to increase statistical power for estimation of CRC risk. It was found that the A carriers have an OR of 1.61 compared with carriers of the CC genotype (95% CI = 1.25–2.06). Additionally, significantly increased risk of CRC was also found in additive model, with per-A-allele OR of 1.60 (95% CI = 1.26–2.02).

**Table 2:** Association between rs961253 and colorectal cancer risk in a Chinese population.

Genotype	Case (N = 641)No. (%)	Control (N = 1037)No. (%)	*P* †	Crude OR (95% CI)	OR (95% CI)‡	*P* ‡
CC	494 (77.1)	875 (84.5)	<0.001	1.00	1.00	
CA	139 (21.7)	157 (15.1)		1.57 (1.22–2.03)	1.56 (1.21–2.01)	0.001
AA	8 (1.2)	4 (0.4)		–	–	–
Dominant model	–		–	1.62 (1.26–2.08)	1.61 (1.25–2.06)	<0.001
Additive model	–		–	1.61 (1.27–2.04)	1.60 (1.26–2.02)	<0.001

Abbreviations: OR, Odds ratio; 95% CI, 95% confidence interval.

† *P* value was calculated by the *χ^2^* test.

‡ Data were estimated by multivariate logistic regression model after adjusting for sex and age.

### Meta-analysis results

#### Study characteristics

As shown in Figure S1, 8 reports were judged to preliminarily fit the inclusion criteria. After detailed evaluation, 2 reports with incomplete data were removed after contacting with authors by e-mail [29], [30]. 3 reports shared the same sample [5], [31], [32], of which, Tomlinson et al. [32] was selected due to the largest sample, although Houlston et al. was the first to suggest the association of rs961253 [5]. Finally, 4 reports plus our case-control study comprising 17 studies of 29859 cases and 29696 controls were included in this meta-analysis [14], [15], [32], [33]. Among these, 14 studies were conducted in European and 3 in Asian (Table S1). Genotypes of rs961253 in controls conformed to Hardy-Weinberg equilibrium for all included studies (*P*>0.01).

#### Frequency of risk allele in control population

There was significant heterogeneity in European group (*P* for heterogeneity <0.001, *I^2^* = 71.1%). The pooled frequency of the A allele was 35.4% (95% CI = 34.6%–36.2%) in European controls under random-effects model, which was markedly higher than that of 8.1% in Asian controls without heterogeneity (95% CI = 7.5%–8.6%, *P* for heterogeneity = 0.449; Figure S2).

#### Overall meta-analysis of rs961253 in associated with CRC

In genotypic model, significant increased risk of CRC was observed for the CA versus CC (OR = 1.14, 95% CI = 1.10–1.18; Table 3) under fixed-effects model (*P* for heterogeneity = 0.167). A marginal heterogeneity was observed in the AA versus CC (*P* for heterogeneity = 0.102) as well as in dominant model (*P* for heterogeneity = 0.099), while significant heterogeneity was found in additive model (*P* for heterogeneity = 0.019). Therefore, random-effects model was applied for the AA genotypic, dominant and additive models, and all of these genetic models conferred significant increased risk of CRC, with ORs of 1.25 (95% CI = 1.16–1.34), 1.17 (95% CI = 1.12–1.22), and 1.13 (95% CI = 1.09–1.18), respectively.

**Table 3:** Meta-analysis of the rs961253 in association with colorectal cancer risk under different genetic models.

Variables	Case/control	Genetic model	OR (95%CI)	*P*	*I^2^* (%)	*P* for heterogeneity
Total (N = 17)	29859/29696	CA versus CC	1.14 (1.10–1.18)	<0.001	24.9	0.167
		AA versus CC	1.25 (1.16–1.34)	<0.001	31.8	0.102
		Dominant model	1.17 (1.12–1.22)	<0.001	32.1	0.099
		Additive model	1.13 (1.09–1.18)	<0.001	46.2	0.019
Ethnicity						
European (N = 14)	26220/25650	CA versus CC	1.12 (1.08–1.16)	<0.001	0.0	0.691
		AA versus CC	1.23 (1.17–1.30)	<0.001	0.0	0.539
		Dominant model	1.14 (1.10–1.18)	<0.001	0.0	0.555
		Additive model	1.11 (1.08–1.14)	<0.001	0.0	0.471
Asian (N = 3)	3639/4046	CA versus CC	1.37 (1.21–1.54)	<0.001	0.0	0.463
		AA versus CC	1.63 (0.44–6.05)	0.465	77.2	0.012
		Dominant model	1.39 (1.21–1.60)	<0.001	22.4	0.276
		Additive model	1.34 (1.20–1.50)	0.001	53.5	0.116

#### Meta-regression analysis and stratified analysis

To explore potential sources of between-study heterogeneity under additive model, meta-regression analysis was performed. A empty regression was firstly run to estimate the baseline value for *tau^2^* (*tau^2^* = 0.0019), and then a series univariate model was conducted by adding single covariates including ethnicity of population, source of control, study type, sample size, and genotyping method. In the univariate analysis, the model including ethnicity reduced the *tau^2^* value to 0, and the adjusted *R^2^*value was 100% (*P* = 0.004), suggesting ethnicity could totally explained the heterogeneity across studies in additive model. Stratified analysis by ethnicity was further performed. In European population, all genetic models showed no evidence of heterogeneity (*P* for heterogeneity>0.1, *I^2^* = 0), and present significantly increased risk of CRC, with ORs of 1.12 (95% CI = 1.08–1.16), 1.23 (95% CI = 1.17–1.30), 1.14 (95% CI = 1.10–1.18), and 1.11 (95% CI = 1.08–1.14) for the genotype CA versus CC, AA versus CC, and dominant and additive models, respectively. In Asian population, only the genotypic model of AA versus CC showed significant heterogeneity (*P* for heterogeneity = 0.012, *I^2^* = 77.2%). All genetic models presented significant increased risk of CRC except for the AA versus CC (Table 3). Additionally, larger effect of the A variant was seen in Asian than that in European.

#### Sensitivity analysis

Since significant heterogeneity across studies was observed for the additive model, we conducted a sensitivity analysis to assess the effect of each study on the pooled estimate under a random-effect model. As shown in Table 4, a series of pooled OR with 95% CI produced repeatedly after removal of each particular study continuously exceed 1.0, and the pooled OR was similar before and after deletion of each study. Similarly results were seen for other genetic models that no single study meaningfully change the pooled ORs, indicating the robust stability of the current results.

**Table 4:** Sensitivity analysis of additive model.

Omitted study	OR (95% CI)	*P* for heterogeneity	*I^2^* (%)
Xiong, 2010 [32]	1.12 (1.08–1.16)	0.104	32.3
von Holst, 2010 [14]	1.14 (1.10–1.19)	0.025	45.5
Tomlinson,2011(UK1) [31]	1.14 (1.09–1.18)	0.013	49.5
Tomlinson, 2011(Scotland1)	1.13 (1.09–1.18)	0.013	49.5
Tomlinson, 2011(UK2)	1.14 (1.09–1.18)	0.013	49.5
Tomlinson, 2011(Scotland2)	1.13 (1.09–1.18)	0.016	48.3
Tomlinson, 2011 (VQ58)	1.14 (1.10–1.19)	0.027	44.8
Tomlinson, 2011 (CCFR)	1.14 (1.09–1.18)	0.015	48.8
Tomlinson, 2011 (Australia)	1.13 (1.09–1.18)	0.014	49.1
Tomlinson, 2011 (Helsinki)	1.13 (1.09–1.17)	0.020	46.9
Tomlinson, 2011 (Cambridge)	1.14 (1.10–1.19)	0.017	48.0
Tomlinson, 2011 (COIN/NBS)	1.14 (1.10–1.19)	0.034	43.3
Tomlinson, 2011 (UK3)	1.14 (1.09–1.19)	0.013	49.5
Tomlinson, 2011 (Scotland3)	1.13 (1.09–1.17)	0.025	45.4
Tomlinson, 2011 (UK4)	1.13 (1.09–1.18)	0.014	49.2
Ho, 2011 [13]	1.13 (1.09–1.18)	0.044	41.2
Zheng, 2011	1.12 (1.09–1.16)	0.150	27.2

#### Cumulative meta-analysis

**Figure 1:**
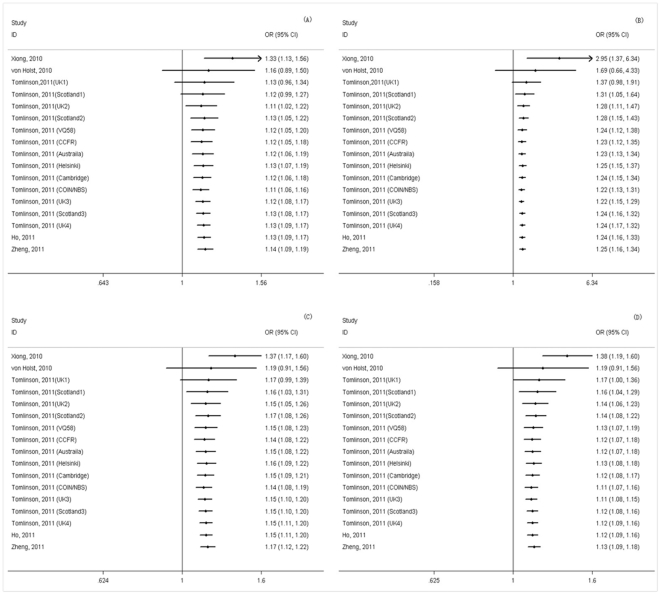
Forest plots of cumulative meta-analysis of rs961253 in association with colorectal cancer by published year under different genetic models. (A) the CA versus CC; (B) the AA versus CC; (C) the dominant model; (D) the additive model.

Cumulative analysis of the association of rs961253 with CRC was conducted via the assortment of studies by publication time. As shown in Figure 1, inclinations toward significant association were evident over time in all genetic models. Further, the 95% CIs became increasingly narrower with each accumulation of more data, suggesting the precision of the estimates was progressively boosted by continual adding more sample.

#### Publication bias

As reflected by the funnel plot and the Egger's test, there was no publication bias in the genotypic models of CA versus CC and AA versus CC and dominant model (*P* for Egger's test = 0.269, 0.198, and 0.187, respectively), whereas a marginally significant publication bias was seen in additive model (*P* for Egger's test = 0.047). Then a trim-and-fill method was implemented under random-effects model. Nevertheless, no trimming was performed and pooled estimate was unchanged, indicating our result was statistically robust.

## Discussion

In this study, we found a significant association between rs961253 and colorectal cancer in the Chinese population. Also, the following meta-analysis pooling data from the current and 16 previously published studies indicated significant association of rs961253 with CRC under genotypic, dominant and additive models. Cumulative analysis further confirmed the significant association, showing the effect of the variant became progressively significant with each accumulation of more data over time. To best our knowledge, this meta-analysis firstly combined published GWA studies and replication studies to reflect a precision effect of rs961253 on the CRC risk.

rs961253 is located at 20p12.3, a region bereft of genes or predicted protein-encoding transcripts. However, BMP2 maps 342 kb telomeric to this locus [5], which is one of initiators of BMP signaling by binding to its corresponding receptors. BMP signaling can suppress the Wnt pathway to ensure a balanced control of intestinal stem cell self-renewal [34]. As reflected by earlier studies, mutations of BMP pathway have been described in juvenile polyposis [35], an inherited syndrome that predisposes to CRC. Recently, tumor suppression role of the BMP signaling has been established, and the BMP pathway has been inactivated in up to 70% of sporadic CRC [36]. Considering all this information, although no function report was concerning to the rs961253, it has been speculated that this locus might alter the BMP signaling transduction by the effect on BMP2 and thus affect CRC incidence [37]. However, after the first GWA study concerning rs961253, the follow-up replications have yielded inconsistent results.

In this study, our data in the Chinese population indicated that increased risk was significantly associated with the CA genotype compared with the CC genotype, and similar significant relationship maintained under the dominant and additive models. For the AA genotype, due to the low frequency in this population, we failed to estimate its precise effect. The following meta-analysis, including 29859 cases and 29696 controls, provided a 100% power for estimating the association between rs961253 and CRC. Results indicated that all genetic models conferred significant increased risk of CRC. However, although there was obvious evidence of between-study heterogeneity for the additive models, the heterogeneity had been totally explained by the ethnicity of study population according to the result of meta-regression analysis. Then stratified analysis by ethnicity was performed. In European population, heterogeneity was removed, and all genetic models of the A variant allele were still significantly associated with increased risk, while all genetic models in Asian also conferred increased risk without evidence of heterogeneity except for the AA genotypic model. After compare male/female ratio, mean age and MAF of risk allele, there were no significant finding between Asian and European except MAF of risk allele. Therefore, the variance of the AA genotypic effect between European and Asian may attribute to different ethnic background characterized by allele frequency difference, with pooled A allele frequencies of 35.4% in European and of 8.1% in Asian. Additionally, ORs of genetic models in Asian were all larger than those in European. In consideration of the inverse relationship between allele frequency and effect size based on purifying selection [38], we proposed that the rs961253 variant might have larger effect in Asian than European.

Ethnicity was also reflected as the main origin of heterogeneity in sensitivity analysis by showing that degree of heterogeneity was reduced after exclusion of single study in Asian. In addition, no single study influenced the overall ORs qualitatively for all genetic models, suggesting the highly stability of the current results. The cumulative analysis provided further support to the current results, indicating that as accumulation of more data over time, the precision of estimates was continuously enhanced and tendency toward significant association was increasingly evident. Publication bias was also comprehensively assessed in this current study. No evidence of publication bias was found in all genetic models except for the additive model as reflected by the funnel plot and Egger's test. We further applied trim-and-fill method to adjust for publication bias. Nevertheless, result showed that meta-analysis with or without the trim-and-fill method did not draw different effect estimate. Taken together, the results of this meta-analysis are sound and reliable.

Despite the clear strength of the current study yielding enough power, some limitations should be addressed. CRC is a complex trait caused by both genetic and environmental factors; however, lacking of environment data limited our further evaluation of gene-environment interaction. Although association between rs961253 and CRC has been confirmed in this study, whether this SNP is causal remained uncertain.

In conclusion, the results from our study in the Chinese population and the meta-analysis combining different ethnicity provided a more accurate depiction of the role of rs961253 in CRC susceptibility, suggesting that the variant of rs961253 was associated with increased risk of CRC, and the variant may yielded larger effect on Asians than Europeans. However, fine-mapping of 20p12.3 region or function analysis should be imposed to identify causal variant.

## Supporting Information

Figure S1 **Flow chart for study selection.**
(TIF)

Figure S2 **Pooled frequency of the A allele in European and Asian controls.**
(TIF)

Table S1 **The characteristics of the studies included in the meta-analysis for the association of rs961253 with CRC.**
(DOC)

Checklist S1 **PRISMA checklist.**
(DOC)
