# Supplementary figures and images for: The SNP rs961253 in 20p12.3 Is Associated with Colorectal Cancer Risk: A Case-Control Study and a Meta-Analysis of the Published Literature
Source: PLoS One. 2012 Apr 11;7(4):e34625. doi: 10.1371/journal.pone.0034625 (PMC3324501; doi:10.1371/journal.pone.0034625)

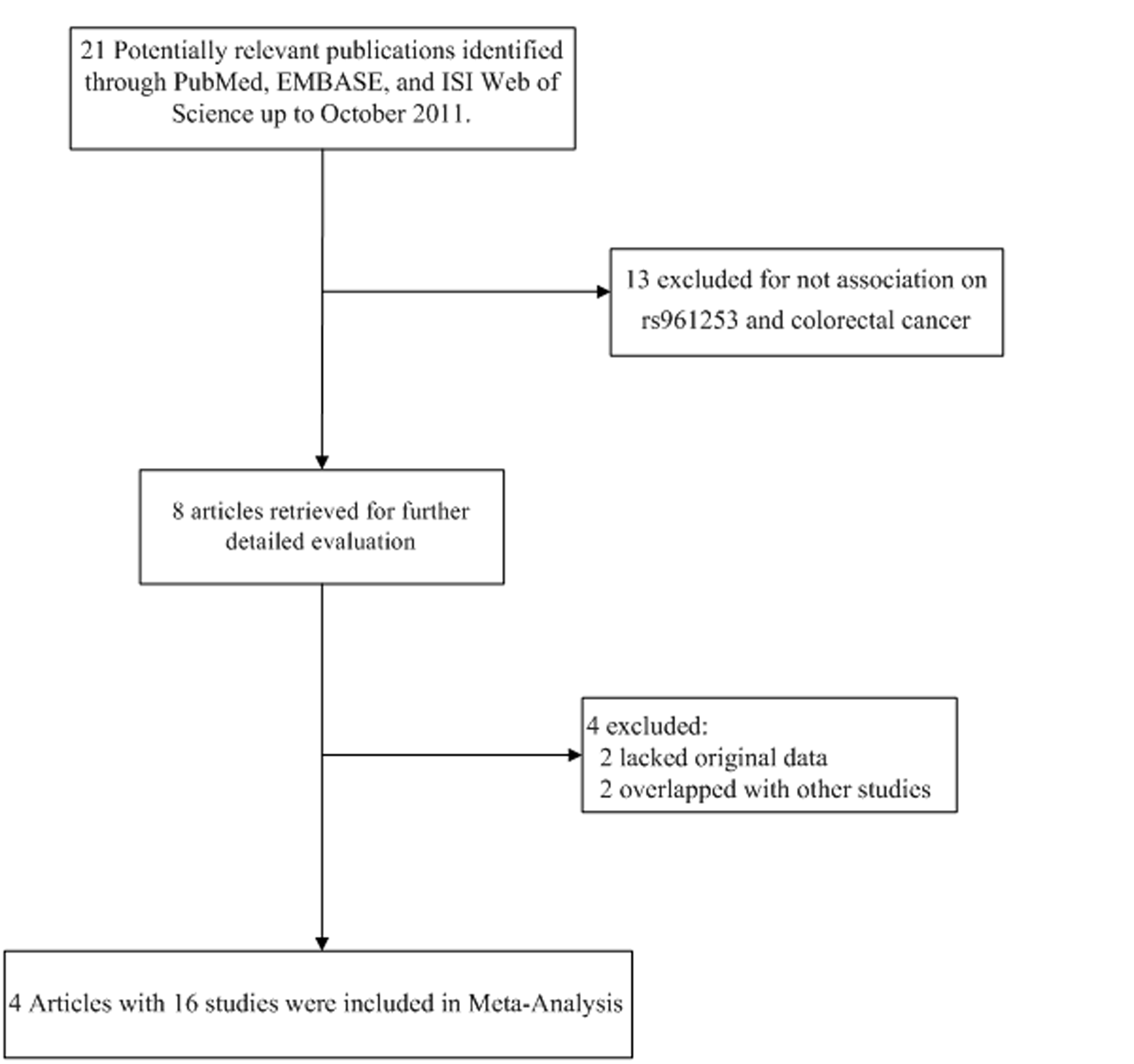

Supplement: Figure S1 — Flow chart for study selection. (TIF) [file pone.0034625.s001.tif]

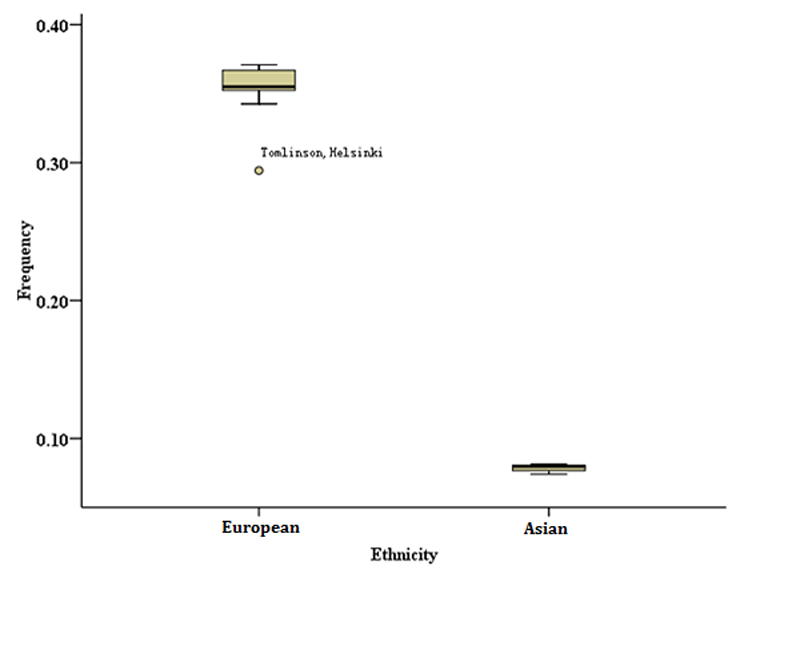

Supplement: Figure S2 — Pooled frequency of the A allele in European and Asian controls. (TIF) [file pone.0034625.s002.tif]
