# Supplementary material for: The SNP rs961253 in 20p12.3 Is Associated with Colorectal Cancer Risk: A Case-Control Study and a Meta-Analysis of the Published Literature
Source: PLoS One. 2012 Apr 11;7(4):e34625. doi: 10.1371/journal.pone.0034625 (PMC3324501; doi:10.1371/journal.pone.0034625)
Supplement: Table S1 — The characteristics of the studies included in the meta-analysis for the association of rs961253 with CRC. (DOC) [file pone.0034625.s003.doc]

Supplementary Table S1. The characteristics of the studies included in the meta-analysis for the association of rs961253 with CRC.

| First author Published year | Country of study | Ethnicity | Control source | Study type | Genotyping method | HWE | Case/Control | Male/female ratio | |  | Mean age | |  | MAF | |
| --- | --- | --- | --- | --- | --- | --- | --- | --- | --- | --- | --- | --- | --- | --- | --- |
|  |  |  |  |  |  |  |  | Case | Control |  | Case | Control |  | Case | Control |
| Xiong, 2010 [32] | China | Asian | Population based | Replication | PCR-RFLP | Yes | 2110/2118 | 1.13 | – |  | 68.6 | 66.3 |  | 0.38 | 0.37 |
| von Holst, 2010 [14] | Swedish | European | Population based | Replication | TaqMan PCR | Yes | 1765/1721 | 1.49 | 1.40 |  | 56.9 | 56.4 |  | 0.11 | 0.08 |
| Tomlinson, 2011 (UK1) [31] | UK | European | Population based | GWA study | Illumina Hap370 | Yes | 921/929 | 0.89 | 0.82 |  | – | – |  | 0.40 | 0.37 |
| Tomlinson, 2011 (Scotland1) [31] | UK | European | Population based | GWA study | Illumina Hap550 | Yes | 977/1001 | 1.05 | 1.04 |  | 49.6 | 51.0 |  | 0.39 | 0.36 |
| Tomlinson, 2011 (UK2) [31] | UK | European | Population based | GWA study | Illumina Infinium | Yes | 2852/2818 | 1.39 | 0.69 |  | 59.3 | 59.8 |  | 0.38 | 0.36 |
| Tomlinson, 2011 (Scotland2) [31] | UK | European | Population based | GWA study | Illumina Infinium | Yes | 2006/2057 | 1.56 | 1.50 |  | 65.8 | 67.9 |  | 0.38 | 0.35 |
| Tomlinson, 2011 (VQ58) [31] | UK | European | Population based | GWA study | Illumina Hap300 | Yes | 1610/2689 | 1.50 | 1.04 |  | 62.5 | – |  | 0.38 | 0.37 |
| Tomlinson, 2011 (CCFR) [31] | USA, Canada | European | Population based | GWA study | Illumina Hap1M | Yes | 1186/996 | – | – |  | – | – |  | 0.37 | 0.35 |
| Tomlinson, 2011 (Australia) [31] | Australia | European | Population based | GWA study | Illumina Hap550 | Yes | 440/438 | – | – |  | – | – |  | 0.39 | 0.35 |
| Tomlinson, 2011 ( Helsinki) [31] | Finland | European | Population based | Replication | KASPar,Taqman, MassARRAY | Yes | 941/814 | – | – |  | – | – |  | 0.34 | 0.29 |
| Tomlinson, 2011 (Cambridge) [31] | UK | European | Population based | Replication | KASPar,Taqman, MassARRAY | Yes | 2016/2144 | 1.27 | 0.72 |  | 59.2 | 57.6 |  | 0.37 | 0.35 |
| Tomlinson, 2011 (COIN/NBS) [31] | UK | European | Population based | Replication | Goldengate | Yes | 2182/2500 | – | 1.00 |  | 63.0 | 53.2 |  | 0.38 | 0.37 |
| Tomlinson, 2011 (UK3) [31] | UK | European | Population based | Replication | KASPar,Taqman, MassARRAY | Yes | 7651/4318 | 1.86 | 0.67 |  | 59.0 | 62.0 |  | 0.38 | 0.35 |
| Tomlinson, 2011 (Scotland3) [31] | UK | European | Population based | Replication | KASPar,Taqman, MassARRAY | Yes | 1103/2182 | 1.00 | 0.89 |  | 53.2 | 51.8 |  | 0.39 | 0.34 |
| Tomlinson, 2011 (UK4) [31] | UK | European | Population based | Replication | KASPar,Taqman, MassARRAY | Yes | 590/1043 | 0.85 | 0.82 |  | 58.3 | 45.1 |  | 0.40 | 0.37 |
| Ho, 2011 [13] | HK, China | Asian | Hospital based | Replication | Sequenom | Yes | 888/891 | 1.38 | – |  | 66.4 | – |  | 0.08 | 0.07 |
| Zheng, 2011 | China | Asian | Hospital based | Replication | TaqMan-PCR | Yes | 641/1037 | 1.50 | 1.50 |  | 56.3 | 57.2 |  | 0.12 | 0.08 |

Abbreviation: HEW, Hardy-Weinberg equilibrium, MAF: minor allele frequency.
